# Pregnancy Outcome in Swiss-Webster Mice Infected With *Chlamydia trachomatis*

**DOI:** 10.1155/S1064744994000165

**Published:** 1994

**Authors:** Bryan T. Oshiro, Jack M. Graham, Jorge D. Blanco, Ibrahim M. Seraj, Karen D. Bishop

**Affiliations:** 1 Department of Obstetrics, Gynecology, and Reproductive Sciences University of Texas Health Science Center Houston TX USA; 2 Department of Obstetrics and Gynecology Loma Linda University Loma Linda CA USA; 3 Department of Perinatology McKay-Dee Hospital Center 3939 Harrison Boulevard Ogden UT 84409 USA

## Abstract

*Objective:* The objective of this study was to observe pregnancy outcomes in mice infected transvaginally with *Chlamydia trachomatis*.

*Methods:* Pregnant mice were inoculated transvaginally with either *C. trachomatis* (CT) or sterile calf serum (CON) on pregnancy day 4. Pregnancy outcomes as well as genital tract histology and culture were compared. Statistical analysis was performed using Fisher's exact test and Student's t-test.

*Results:* Twenty-four of 26 CT mice had positive uterine cultures for *C. trachomatis.* Inflammation occurred in 9 (34.6%) (*P* = 0.002, 95% confidence interval = 1.7–3.5) and intrauterine fetal demise occurred in 5 (19.2%) (*P* = 0.05, 95% confidence interval = 1.6–2.9) of CT mice. No mice in the CON group (0/24) had positive uterine cultures, developed inflammation, or experienced intrauterine fetal demise.

*Conclusions: * Lower genital tract chlamydial infection is associated with intrauterine fetal demise in Swiss-Webster mice.


Infectious Diseases in Obstetrics and Gynecology 1:242–245 (1994)
© 1994 Wiley-Liss, Inc.

### Pregnancy Outcome in Swiss-Webster Mice Infected With *Chlamydia trachomatis*

**Bryan T. Oshiro, Jack M. Graham, Jorge D. Blanco, Ibrahim M. Seraj, and Karen D. Bishop**

*Department of Obstetrics, Gynecology, and Reproductive Sciences, University of Texas Health Science Center, Houston, TX (B.T.O., J.M.G., J.D.B., K.D.B.), and Department of Obstetrics and Gynecology, Loma Linda University, Loma Linda, CA (I.M.S.)*

### Abstract

***Objective:*** **The objective of this study was to observe pregnancy outcomes in mice infected transvaginally with *Chlamydia trachomatis*.**

***Methods:*** **Pregnant mice were inoculated transvaginally with either *C. trachomatis* (CT) or sterile calf serum (CON) on pregnancy day 4. Pregnancy outcomes as well as genital tract histology and culture were compared. Statistical analysis was performed using Fisher's exact test and Student's t-test.**

***Results:*** **Twenty-four of 26 CT mice had positive uterine cultures for *C. trachomatis*. Inflammation occurred in 9 (34.6%) ($P$ = 0.002, 95% confidence interval = 1.7–3.5) and intrauterine fetal demise occurred in 5 (19.2%) ($P$ = 0.05, 95% confidence interval = 1.6–2.9) of CT mice. No mice in the CON group (0/24) had positive uterine cultures, developed inflammation, or experienced intrauterine fetal demise.**

***Conclusions:*** **Lower genital tract chlamydial infection is associated with intrauterine fetal demise in Swiss-Webster mice.** © 1994 Wiley-Liss, Inc.

Key words

*Chlamydia trachomatis,* fetal demise, Swiss-Webster mice

*Chlamydia trachomatis* is recognized as the most common sexually transmitted organism, with over 4 million cases reported annually.[1] *C. trachomatis* is responsible for causing inclusion conjunctivitis and pneumonia in the neonate and is the leading cause of preventable blindness in the world.[2]

Although *C. trachomatis* has clearly been shown to cause mucopurulent cervicitis,[3] acute salpingitis,[4] and neonatal disease, its effect on pregnancy outcome remains controversial. Martin and colleagues[5] reported that the incidence of prematurity and perinatal death was higher in women with a *C. trachomatis* infection identified before 20 weeks. In contrast, other authors[6,7] have not found an association between *C. trachomatis* infection and adverse pregnancy outcome.

The Swiss-Webster mouse has been used extensively to study the effects of *C. trachomatis* on the female genital tract.[8–10] Rank et al.[11] have reported infecting mice transvaginally with *C. trachomatis*. However, to our knowledge, an animal model has not been developed to study the effects of *C. trachomatis* lower genital tract infection on pregnancy outcome.

To determine whether adverse pregnancy outcome is associated with *C. trachomatis* genital tract infection in an animal model, we studied the effect of lower genital tract *C. trachomatis* infection in pregnant Swiss-Webster mice.

**Address correspondence/reprint requests to Dr. Bryan T. Oshiro, Department of Perinatology, McKay-Dee Hospital Center, 3939 Harrison Boulevard, Ogden, UT 84409.**

Received November 15, 1993

Accepted March 11, 1994

### SUBJECTS AND METHODS

### Mice

Seventy-six 6–8-week-old female Swiss-Webster mice (Sasco, Inc., Omaha, NE) with unlimited access to food and water were placed 2 per cage with a male of established fertility over a 24-h period. The mice were then separated into individual cages. Pregnancy was confirmed by the presence of a copulation plug and later by palpation of a pregnant uterus. Twenty-six non-pregnant mice were removed from the study. This experiment was approved by the Animal Welfare Committee of the University of Texas Health Science Center at Houston, and all practices were in accordance with this committee's guidelines.

### Chlamydia

The mouse pneumonitis strain of *C. trachomatis* ATCC BR-123 was reconstituted to a concentration of approximately $1 \times 10^6$ inclusion-forming units/ml in sterile calf serum as described by Blanco et al.[10]

### Experimental Design

The female mice were separated into 2 groups: a group for chlamydial (CT) inoculation and a control group (CON). The inoculation technique used in this experiment has been reported by Rank et al.[11] On pregnancy day 4, the CT group was inoculated transvaginally with chlamydia suspended in 50 μl of sterile calf serum. The CON group was similarly inoculated with 50 μl of sterile calf serum. On pregnancy day 7, lower genital tract cultures were obtained on all mice using a dacron-tipped aluminum swab which was placed in chlamydia transport media and immediately frozen at −70°C. The mice were observed daily for signs of clinical infection or preterm delivery. The mice were sacrificed and a laparotomy was performed on the day of delivery. If delivery did not occur by the normal date of confinement (day 21), the observation period was extended to pregnancy day 24 at which time all undelivered mice were sacrificed and explored. The abdominal cavity and reproductive tract were grossly examined for evidence of inflammation and the uterus was removed. The distal most portion of the uterus was transected and placed in chlamydia transport media and frozen at −70°C. The remainder of the uterus was placed in 10% formalin. The pups were counted and weighed.

### Culture

The lower genital tract culture swab specimens were allowed to thaw at room temperature and vortexed for 15 min. The uterine tissue was also thawed, then morcelated and vortexed. The supernatants were removed and placed on McCoy cells which were previously washed with $Mg^{2+}$, $Ca^{2+}$, and phosphate-buffered saline. The cells were then centrifuged for 1 h at 3,400*g* and incubated at 37°C for 48 h. A second-pass culture was performed on all samples. The culture technique is standard and has been described elsewhere.[12] The cells were stained with a chlamydia-specific fluorescent antibody stain (Bartels Diagnostic Division, McGaw Park, IL) and examined by indirect fluorescent microscopy in a blinded fashion.

### Histology

The formalin-fixed tissue was placed in paraffin blocks, sliced, stained with hematoxylin and eosin, and mounted on glass slides. The examiner was blinded as to whether the specimens originated from CT or CON animals. The tissue was classified as inflamed if plasma cells or a polymorphonuclear infiltrate were present on examination of the slides.

### Statistical Analysis

Statistical analysis was performed by Fisher's exact test and Student's t-test where appropriate. $P < 0.05$ was considered statistically significant.

### RESULTS

Twenty-six mice in the CT group and 24 mice in the CON group became pregnant and were included in the study. All 26 (100%) of the CT mice had positive lower genital tract swab cultures for chlamydia on day 7. Uterine tissue cultures were positive in 24/26 (92.3%) of CT mice. Inflammation of the uterine tissues and intrauterine fetal death were significantly increased in the CT mice (Table 1). In the 5 pregnancies in which fetal death occurred, all of the pups in the litters were dead. Placental histology could not be determined in each pregnancy affected by intrauterine fetal demise because of the various stages of resorption of the pregnancy products, but extensive inflammation

**TABLE 1. Adverse pregnancy outcome data in mice infected with *C. trachomatis***

| | CT (n = 26) | CON (n = 24) | *P* | Relative risk | 95% Confidence interval |
|---|---|---|---|---|---|
| Inflammation | 9 (35%) | 0 (0%) | 0.002 | 2.4 | 1.7–3.5 |
| Fetal deaths | 5 (19%) | 0 (0%) | 0.05 | 2.2 | 1.6–2.9 |

**TABLE 2. Delivery data in mice with liveborn pups[a]**

| | CT (n = 21) | CON (n= 24) | Significance |
|---|---|---|---|
| No. live pup-mouse | 9.0 ± 1.7 | 8.9 ± 2.2 | NS |
| Pup weight (g) | 1.59 ± 0.24 | 1.59 ± 0.15 | NS |
| Pregnancy days | 21.6 ± 1.1 | 21.2 ± 1.6 | NS |

[a]Values are mean ± 1 SD. NS = not significant.

was observed in the placentas that were examined. In 4 of the pregnancies in which fetal death occurred, the gestational sacs appeared to be intact. Because of advanced resorption of pregnancy products in the 5th pregnancy, the status of the sacs could not be determined. There were no instances of preterm delivery in the CT group, and none of the mice with intrauterine fetal death delivered their pups by the end of the normal confinement period. The fetal deaths occurred only in the group of mice with uterine inflammation. None of the mice in the CON group had positive cultures, evidence of an inflammatory reaction in uterine tissues, or intrauterine fetal deaths. Table 2 shows the pregnancy outcome data in mice with liveborn pups. No significant difference was found in the mean duration of pregnancy, the mean number of live pups born per mouse, or the mean weight of the liveborn pups between the CT and the CON groups if fetal death did not occur.

### DISCUSSION

The role of *C. trachomatis* in adverse pregnancy outcomes is controversial. In humans, several studies have not shown an association between *C. trachomatis* infection and adverse pregnancy outcomes.[6,7,13–18] However, others have implicated *C. trachomatis* in premature rupture of the membranes, preterm birth, low birth weight, intraamniotic infection, and stillbirth.[5,19–24]

One of the first studies to suggest that *C. trachomatis* was associated with adverse pregnancy outcomes was performed by Martin and colleagues[5] in 1982. In their study, 18 of 268 (6.7%) pregnant women screened before 20 weeks gestation were positive for *C. trachomatis*. Twenty-eight percent of the chlamydia-positive patients delivered prematurely compared with 6% of the chlamydia-negative patients. Most significantly, the perinatal mortality in the infected group was 33% compared with 0.4% in the uninfected group. Gravett et al.[19] found that chlamydial infection of the cervix was associated with low birth weight, premature rupture of the membranes, and preterm birth. Martius et al.[20] and The Johns Hopkins Study Group for Cervicitis and Adverse Pregnancy Outcomes[21] also found that cervical infection with *C. trachomatis* was significantly associated with preterm birth.

Chlamydial infections of the cervix may ascend and infect the membranes and fetus. Thorp et al.[22] reported a case of fetal death attributed to an intraamniotic chlamydial infection across intact membranes. Although chlamydia was never cultured from the fetus, the formalin-fixed lung tissue was highly positive for fluorescent antibody against chlamydia. Other authors[23,24] have also reported in utero chlamydial infection across intact membranes.

In this study, we found that lower genital tract infection with chlamydia caused infection of uterine tissues across intact membranes and furthermore was significantly associated with intrauterine demise in Swiss-Webster mice. We also found that, in contradistinction to humans, intrauterine or intraamniotic chlamydial infection in mice did not result in premature rupture of the membranes or preterm delivery. Rather, the affected pregnancies were retained past their normal term of gestation.

With the exception of the study by Martin and colleagues,[5] human studies have not found an increase in stillbirth associated with chlamydial infection in pregnancy. Many other studies looking at the effect of *C. trachomatis* in pregnant women have had small sample sizes and did not specifically look at stillbirths as an outcome variable. We have shown that *C. trachomatis* is capable of causing intrauterine fetal death in Swiss-Webster mice. These results may not correlate to adverse pregnancy outcomes in humans, but it would be interesting to determine if *C. trachomatis* is a significant cause of stillbirth in human pregnancy as we have demonstrated in the Swiss-Webster mice.

### REFERENCES

1. Centers for Disease Control: *Chlamydia trachomatis* infections: Policy guidelines for prevention and control. MMWR 34(Suppl 3):53s–74s, 1985.
2. Schachter J, Hanna L, Hill EC, Massad S, Sheppard CW, Conte JE, Meyer KF: Are chlamydial infections the most prevalent venereal disease? JAMA 231:1252–1255, 1975.
3. Brunham RC, Paavonen J, Stevens CE, et al.: Mucopurulent cervicitis: The ignored counterpart in women of urethritis in men. N Engl J Med 311:1–6, 1984.
4. Sweet RL: Chlamydial salpingitis and infertility. Fertil Steril 38:530–533, 1982.
5. Martin DH, Koutsky K, Eschenbach DA, et al.: Prematurity and perinatal mortality in pregnancies complicated by maternal *Chlamydia trachomatis* infections. JAMA 247: 1585–1588, 1982.
6. Harrison HR, Alexander ER, Weinstein L, et al.: Cervical *Chlamydia trachomatis* and mycoplasmal infections in pregnancy. Epidemiology and outcomes. JAMA 250: 1721–1727, 1983.
7. Sweet RL, Landers DV, Walker C, Schachter J: *Chlamydia trachomatis* infection and pregnancy outcome. Am J Obstet Gynecol 156:824–833, 1987.
8. Swenson CE, Donegan E, Schachter J: *Chlamydia trachomatis*-induced salpingitis in mice. J Infect Dis 148: 1101–1107, 1983.
9. Patton DL, Landers DV, Schachler J: Experimental *Chlamydia trachomatis* salpingitis in mice: Initial studies on characterization of the leukocyte response to chlamydial infection. J Infect Dis 159:1105–1110, 1989.
10. Blanco JD, Patterson RM, Ramzy I, Turner T: Clindamycin and ibuprofen effects on chlamydial salpingitis in mice. Sex Trans Dis 16:192–194, 1989.
11. Rank RG, Ramsey KH, Hough AJ: Antibody-mediated modulation of arthritis induced by chlamydia. Am J Pathol 132:372–381, 1988.
12. Ripa KT, Mardh P-A: Cultivation of *Chlamydia trachomatis* in cycloheximide-treated McCoy cells. J Clin Microbiol 6:328–331, 1977.
13. Heggie A, Lumicao GG, Stuart LA, Gyves MT: *Chlamydia trachomatis* infection in mothers and infants: A prospective study. Am J Dis Child 135:507–511, 1981.
14. Berman SM, Harrison HR, Boyce WT, Haffner WJ, Lewis M, Arthur JB: Low birth weight, prematurity, and post-partum endometritis. Association with prenatal cervical *Mycoplasma hominis* and *Chlamydia trachomatis* infection. JAMA 257:1189–1194, 1987.
15. Ross SM, Windson IM, Robins-Browne RM, Ballard RC, Adhikari M, Fenn DB: Microbiological studies during the perinatal period: An attempt to correlate selected bacterial and viral infections with intrauterine deaths and preterm labor. S Afr Med J 66:598–603, 1984.
16. FitzSimmons J, Callahan C, Shanahan B, Jungkind D: Chlamydia infection in pregnancy. J Reprod Med 31:19–22, 1986.
17. Hardy PH, Nell EE, Spence MR, et al.: Prevalence of six sexually transmitted disease agents among pregnant inner-city adolescents and pregnancy outcome. Lancet 2:333–337, 1984.
18. Thompson S, Lopez B, Wong K-H, et al.: A prospective study of chlamydia and mycoplasma infections during pregnancy: Relation to pregnancy outcome and maternal morbidity. In Mardh P-A, et al. (eds): Chlamydia Infections. Amsterdam: Elsevier Biomedial Press, pp 15–18, 1982.
19. Gravett, MG, Nelson HP, DeRouen T, Critchlow C, Eschenbach DA, Holmes KK: Independent associations of bacterial vaginosis and *Chlamydia trachomatis* infection with adverse pregnancy outcome. JAMA 256:1899–1903, 1986.
20. Martius J, Krohn M, Hillier S, et al.: Relationships of vaginal *Lactobacillus* species, cervical *Chlamydia trachomatis*, and bacterial vaginosis to preterm birth. Obstet Gynecol 71:89–95, 1988.
21. The Johns Hopkins Study Group for Cervicitis and Adverse Pregnancy Outcomes: Association of *Chlamydia trachomatis* and *Mycoplasma hominis* with intrauterine growth retardation and preterm delivery. Am J Epidemiol 129: 1247–1251, 1989.
22. Thorp JM Jr, Katz VL, Fowler LJ, Kurtzman JT, Bowes WA Jr: Fetal death from chlamydial infection across intact amniotic membranes. Am J Obstet Gynecol 161: 1245–1246, 1989.
23. Givner LB, Rennels MB, Woodward CL, Huang S-W: *Chlamydia trachomatis* infection in infant delivered by cesarean section. Pediatrics 68:420–421, 1981.
24. La Scolea LJ, Paroski JS, Burzynski L, Faden HS: *Chlamydia trachomatis* infection in infants delivered by cesarean section. Clin Pediatr 23:118–120, 1984.
